# Evaluation of two environmental DNA (eDNA) approaches for monitoring hemlock woolly adelgid (HWA)

**DOI:** 10.17912/micropub.biology.001346

**Published:** 2025-01-31

**Authors:** Kathryn Geller, Charlyn Partridge

**Affiliations:** 1 Annis Water Resources Institute, Grand Valley State University

## Abstract

Eastern hemlocks ( *Tsuga canadensis)* are experiencing significant mortality due to the invasive insect, hemlock wooly adelgid (HWA) ( *Adelges tsuga)* . Common monitoring methods for HWA detection include visual assessment of hemlock trees for the presence of ovisac material. However, this method requires time and resources to survey large areas. We compared two methods, ball sampling coupled with genetic analysis and passive environmental DNA (eDNA) sampling, and evaluated their ability to detect HWA in infested and high-risk areas using qPCR. We found that the two methods were equally effective at detecting HWA and could serve as supplemental methods for early HWA monitoring.

**Figure 1. Comparison of eDNA traps and ball sampling for HWA detection:**
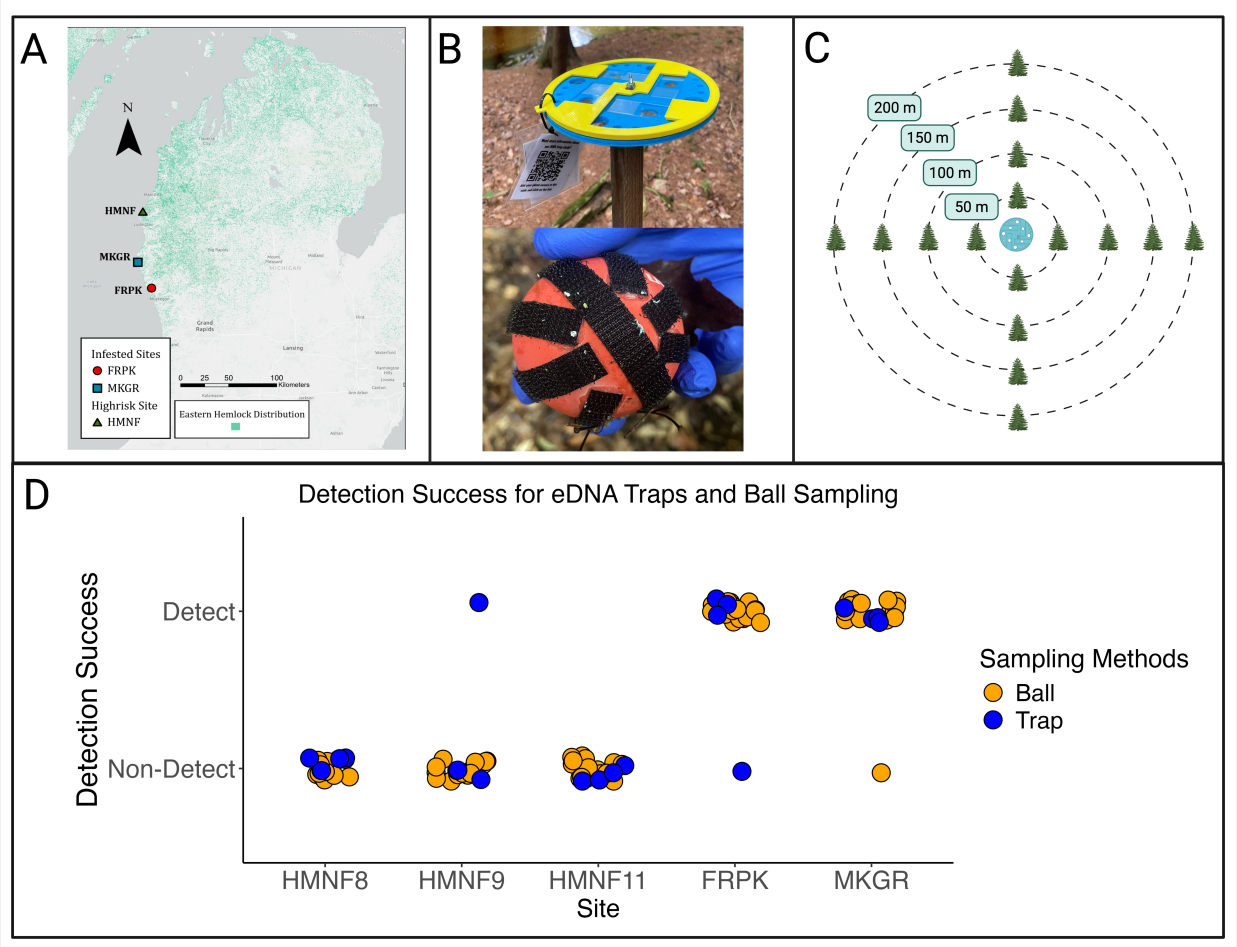
(A) Map depicting location of the study sites within Michigan, USA. Duck Creek National Area in Fruitland, MI (FRPK), and Muskegon Conservation District (MKGR) in Muskegon County, MI are sites with known HWA infestations. Huron-Manistee National Forest (HMNF) sites are high-risk sites in Mason County, MI. (B) eDNA trap (top) and racquetball (bottom) used for ball sampling. The white material on the racquetball is ovisac material collected during sampling. (C) Ball sampling design. Using the eDNA trap as a centroid, we created a 50, 100, 150 and 200 m radius around each trap. Sixteen trees around each eDNA trap were sampled, and this included four trees in each cardinal direction (N, S, E, W). (D) Detection success from the eDNA traps, and ball sampling methods across the study sites. HMNF sites 8, 9, 11 represent three distinct locations within the national forest. The trap samples represent one eDNA trap per site sampled over four collection periods. The ball samples represent 16 trees sampled at each site.

## Description

Hemlock woolly adelgid (HWA) ( *Adelges tsugae* , Annand) is a forest pest that is invasive to eastern regions of North America. Originally native to Japan, this aphid-like insect is now present in at least 20 states across the United States and two provinces in Canada. Since it was originally introduced it has caused a significant decline in Eastern hemlocks ( *Tsuga canadensis,* (L.) Carrière), the primary host in its invasive range. This decline is due to HWA feeding on hemlock nutrients, resulting in a loss of foliage and a combination of other internal and biochemical changes within the tree (Havill et al., 2011) . Currently Eastern hemlocks have shown limited resistance to HWA (Oten et al., 2014) , and once infested, these trees typically die within 5-15 years (Ellison et al., 2010) . The loss of hemlocks can significantly shift the species composition in these areas, and drive landscape and ecosystem-level changes (Orwig and Foster, 1998; Stadler et al., 2006; Spaulding and Rieske, 2010) .

HWA was discovered in west Michigan in 2015 in Allegan County. Since that time, eight other Michigan counties have a confirmed HWA presence: Muskegon, Ottawa, Oceana, Mason, Benzie, Leelanau, Antrim and Washtenaw. Michigan is home to 170 million Eastern hemlocks, so managing this invasive pest is vital to securing the health of Michigan forests. Current HWA monitoring methods within Michigan primarily consist of sending survey crews to look for the presence of HWA ovisac material within hemlock trees. HWA ovisacs consist of white material that is secreted by the adults while feeding, which creates small white woolly ‘balls’ on the base of hemlock needles (McClure, 1987) . This form of monitoring can potentially miss infestations if they are low in density or begin at the top of the hemlock canopy (Evans and Gregoire, 2007) , which can be hard to visually assess.

One approach that can be used to aid with these visual monitoring efforts is the use of environmental DNA (eDNA). eDNA is detectable traces of DNA that organisms leave in their environment. They consist of particles such as tissue fragments or excrement and can be found in water, soil, and air. Airborne eDNA can be a powerful tool for analyzing terrestrial ecosystems (Johnson et al., 2021; Clare et al., 2022; Lynggard et al., 2022; Roger et al., 2022; Johnson et al., 2023; Johnson and Barnes, 2024; Lynggard et al., 2024) and methods based on these approaches have successfully been used to monitor for the presence of HWA (Sanders et al., 2021, Kirtane et al., 2022) . We compared two different methods for HWA monitoring that incorporate eDNA approaches. The first method included passive eDNA traps that were deployed in our sites. The second method included a ball sampling approach, where Velcro covered balls were shot through the upper canopy of hemlocks (Fidgen et al., 2016; Fidgen et al., 2019; Fidgen et al., 2021) , and the material captured was subjected to genetic analysis. We evaluated the efficiency of these methods in two HWA infested sites and three high-risk areas with no known current infestation in Michigan to see if they could help with early monitoring of HWA.

We found consistent results in terms of HWA detections in infested and high-risk sites between the two sampling methods. There was no significant difference associated with detection success across our sites between the passive eDNA traps or the ball samples based on our qPCR results (z = 0.012, p = 0.99). At one of our infested sites in Fruitport, Michigan, (FRPK), the majority of samples from both methods resulted in a positive detection of HWA, except for one eDNA trap sample. Our second infested site that is managed by the Muskegon Conservation District (MKGR) had similar results. Most of the samples from the eDNA trap and ball samples indicated positive detections, with one ball sample indicating no detection. At the high-risk sites in Huron-Manistee National Forest (HMNF8 and HMNF11), neither ball samples nor trap samples indicated a positive detection. Site HMNF9 had mostly no detections, although one eDNA trap sample indicated a positive detection ( Figure 1C ).

There are many benefits of combining eDNA approaches with current monitoring tools. First, the passive eDNA trap allows for continuous monitoring within a given area over a set period of time. In this study, we surveyed high-risk areas for two months using the eDNA traps, which can increase the detection probability for these high-risk sites. This is different from the current visual surveys, where sites are often only surveyed once annually and the goal is to sample many trees, even though thorough observation of each tree may be lost. The addition of genetic analysis for both methods also helps ensure that HWA is being identified correctly. Other adelgids, such as pine adelgids ( *Pineus strobi* ), also live in the same location as HWA in our monitoring areas. Without taxonomic expertise, it can be hard to tell these apart visually and genetic analysis can help confirm that an adelgid collected is HWA. Similarly, for the ball sampling, there are other organisms that can produce white material, and ovisacs may be confused with spider’s egg sacs or mealybugs (Family Pseudococcidae) by non-experts. Incorporation of genetic analysis for the ball surveys allows managers to be confident that the white material they observed are HWA ovisacs.

Our eDNA trap did pick up a positive detection of HWA at site HMNF9, a site that has no prior history of HWA being present. We alerted forest managers in Huron-Manistee National Forest and provided the GPS location of the trap, which allowed them to focus their visual surveys in this area. An infested tree was later confirmed near the eDNA trap (K. Konan, personal communication) and current treatment plans are in place.

Even though our eDNA trap at site HMNF9 detected HWA, we did not detect any HWA in the trees around the trap from ball sampling. There could be many reasons why our eDNA trap method picked up this positive in a high-risk site and not our ball sampling. The findings of Fidgen et al. (2021) recommend sampling a singular tree at least 20 times for a 0.75 probability of visual detection of ovisac material. However, we only sampled each tree 10 times. Other studies by Fidgen et al. (2016, 2019, 2021) also suggested sample sizes exceeding 100 sampled trees for high detection probability. This is significantly more than the number of trees we sampled (16 trees per site), due to the time and personnel constraints of this project. Management teams should consider this difference when deciding to implement this method and would benefit from sampling more trees per site. Another issue we encountered was that due to the nature of the environment and the weather conditions at the time of sampling, our balls collected sand, soil and other leaf litter when they landed on the ground. This debris most likely contained compounds that are known PCR inhibitors (i.e., humic acid (Tebbe and Vahje, 1993) ) that could have prevented a positive detection. If this was the case, however, we would also expect to see little to no detection of HWA for the ball samples at our infested sites (MKGR and FRPK) due to the inhibitors. Yet, all but one of our ball samples at these sites detected HWA, suggesting that PCR inhibitors are likely not a significant issue for this method. There were also instances where we did not detect HWA DNA on one of our eDNA traps, even in infested regions. For example, our trap in FRPK did not detect HWA for one of the four sampling periods during its deployment. Trap sampling is a more passive and opportunistic approach compared to ball sampling. Since only approximately ¼ of each slide contains petroleum jelly this is a relatively small surface area for capturing adelgid material, resulting in some detections in a site being missed. However, this trap in FRPK did detect the presence of HWA during the other three collecting periods. Because the traps are passive samplers, we do recommend deploying them for an extended period of time (early June – end of July) to increase the probability of detection success (Sanders et al., 2023) .

The use of genetic monitoring with eDNA is a powerful tool capable of helping forest managers in controlling the spread of invasive species. Though airborne eDNA is a relatively new tool, when coupled with other monitoring methods it can help with the detection of small forest pests, such as HWA. Implementation of these methods could help balance the need for long-term monitoring across large geographic areas, while allowing vital personnel to be reserved for treating infested areas to help limit the spread of HWA into new areas.

## Methods

Site Locations

We selected three study areas within Muskegon, Oceana, and Mason Counties in Michigan for this study ( Figure 1A ). Of these areas, two are known to have current HWA infestations. Our first infested area was a moderately infested area near Bigsbie Lake, Michigan: a property managed by Muskegon Conservation District (MKGR). This area received treatment for its HWA infestation in 2021. Duck Creek Natural Area in Fruitport, Michigan (FRPK) is our second infested area; this area is a highly infested area that is currently untreated. Infestation levels for these sites were based on a separate study that used a random branch sampling approach to evaluate the number of sistentes present on 100 new growth needles across 10 trees per site (Dunham, 2024) . In addition to these two infested areas, we sampled multiple locations within an uninfested area in Huron Manistee National Forest (HMNF). We sampled three locations within HMNF that are considered high-risk, meaning there is no current infestation, but there is a high likelihood they may become infested. Two locations are along a well-used hiking trail in Nordhouse Dunes Wilderness Area in Free Soil, Michigan (HMNF8 & HMNF9). The third location in Huron Manistee National Forest (HMNF11) is across from private property that has a known HWA infestation, which was treated in 2022. These locations were selected so we could compare success rates between areas where there is no known infestation to infested areas.

eDNA Trap Sampling

The passive eDNA traps are modified from those originally proposed by Sanders et al. (2023). These traps consist of two parts. The base, which is a 3D printed disk 180 cm in diameter, is made to hold 4 microscope slides coated in petroleum jelly. The top is a ring with tabs that hold these microscope slides in place. The ring and the disk are secured together by a screw and secured atop a 1.5 m wooden pole ( Figure 1B ). We monitored one trap per site, and previous studies have shown that similar traps are able to effectively monitor approximately 1-acre of land (Sanders et al., 2023) . The traps were monitored from the end of May – mid June and the microscope slides on the eDNA traps were exchanged every two weeks, for a total of four collecting periods per site. We stored the slides in 50 mL Falcon tubes, and placed them on ice for transport to Robert B. Annis Water Resources Institute. The slides were then stored at 4ºC until they could be processed.

The petroleum jelly was scraped and transferred to a Zymo bead-bashing tube (bead size 2.0 mm and 0.1 mm) (Zymo, Irvine, CA). We conducted the DNA extractions with Zymo Quick-DNA Tissue Insect Microprep kits (Zymo) using a few modifications. After the petroleum-jelly from each of the slides was placed into the tube, we added the bead-bashing buffer. To separate out the captured material from the petroleum jelly and homogenize the sample, we put the samples through a series of heating and bead-bashing cycles. The samples were initially placed on a heat block at 65ºC for 15 min, while vortexing every 5 minutes to ensure that our petroleum jelly was liquid before lysis. We then homogenized the samples using a tissue-lyser set to 50 rotation per second for 2 minutes. This heat/lyse cycle was performed five times so that each sample was homogenized for at least 10 minutes. After the last heating period, the samples were centrifuged at 11,000 rpm for 1 minute. This resulted in complete separation of any captured material from the petroleum jelly. Using a clean toothpick, we made a small hole in the top layer of petroleum jelly and moved the liquid to a Zymo-Spin III F filter column. The remainder of the extraction protocol proceeded as outlined by the manufacturer. All extracted DNA was stored at -20ºC until we performed the qPCR analysis.

Ball Sampling

Our ball sampling method was modified from Fidgen et al. (2019), where we used the model *FV*_50_ balls for sampling. This method requires using a slingshot to shoot a velcro-covered racquetball through the branches of a singular hemlock tree. The theory being, if there are ovisacs present in the upper canopy, the velcro hooks will pick up the ovisac material which can then be seen upon observation ( Figure 1B ).

To evaluate how ball sampling compared to our eDNA trap sampling, ball sampling targeted the same areas as the eDNA trap samples. Using the eDNA traps as a centroid point, we created a 50, 100, 150 and 200 m radius around each trap. Sixteen trees around each eDNA trap were sampled, and this included four trees in each cardinal direction (N, S, E, W) with each at a distance of 50, 100, 150 or 200 m away from the trap ( Figure 1C ). If there was not a hemlock tree at the exact distance, we sampled the closest hemlock tree in the immediate area. We used a slingshot to pass a ball ten times through the branches of each of the targeted hemlock trees, ensuring the ball hit at least one branch for each of the 10 passes. After the 10 passes, we collected the ball from the ground and checked the ball for the presence of ovisac material. The ball was then placed in a sterile whirl-pak and sealed. To reduce the risk of contamination we wore sterile gloves, changed gloves between trees, and sanitized the slingshot before handling balls for a new tree. We also used new, clean balls for each tree at each sampling site. Sampling was done over the course of two days (June 27 and June 29, 2023), with all HMNF sites being sampled on the first day and MKGR/FRPK being sampled on the second day.

After sampling, the sealed whirl-paks with the balls were brought to the lab and filled with 150 mL of lab-grade water and shaken for 30 seconds, before being stored in a walk-in refrigerator at 4ºC. Balls were left at 4ºC between 24-48 hours prior to filtration (due to time constraints with filtration). Balls were taken out of the walk-in refrigerator in batches of six and were hand-shaken for 30 seconds before being placed on a temperature controlled shaker table for 30 minutes. After 30 minutes, the water was filtered through a 0.4 µm polycarbonate membrane filter (Pall Corporation, Port Washington, NY). The bags and balls were then rinsed with 20 mL of lab grade water which was also filtered. Once dry, filters were folded and placed in Zymo bead-bashing tubes (bead size 2.0 mm and 0.1 mm) to be extracted.

Similar to the eDNA trap samples, we extracted the DNA using Zymo Quick-DNA Tissue Insect Microprep kits (Zymo) using a few modifications. The bead-bashing buffer was added to the bead-bashing tubes with the filter, and then placed in a tissue-lyser (ThermoFisher, Waltham, MA). The beads were homogenized for 10 minutes at 50 rotations per second. The remainder of the extraction protocol followed the manufacturer's instructions. The extracted DNA was stored at -20ºC until we performed the qPCR analysis.

qPCR analysis

We ran qPCR analysis to determine the presence or absence of HWA on the eDNA traps or ball samples using a modified version of the qPCR protocol developed by Kirtane et al. (2021). The qPCR reactions consisted of 1X Taqman Environmental MasterMix (Thermo Fisher, Waltham, MA), 0.6 μM final concentration of the forward and reverse primer, 0.3 μM final concentration for the probe (Fam labeled with a Zen/Iowa Black ^TM^ FQ double quencher) (Integrated DNA Technologies, Coralville, IA), 0.2 mg/mL final concentration of bovine serum albumin (BSA), and 2 µL of DNA. All reactions were run in a 20 µL volume.

The thermal cycling conditions included a pre-holding stage at 60ºC for 30 seconds, followed by a holding stage of 50ºC for 2 minutes and then 95ºC for 10 minutes. The cycling stage consisted of 95ºC for 15 seconds, followed by 60ºC for 1 minute for a total of 40 cycles. All samples were run in triplicate, along with a positive control and a no template control. The positive control consisted of a synthetic G-block that included the HWA target sequence (Kirtane et al., 2021) . Samples that amplified for all three replicates were recorded as ‘HWA detected’ and samples that did not amplify were recorded as ‘HWA not detected’.

To evaluate statistical differences in HWA detection success between the eDNA passive traps and ball samples across sites, we ran a generalized linear model, with a binomial distribution. Statistical analyses were performed with the R version 4.2.3.

## Data Availability

Description: Detect and non-detect data for trap and ball sampling.. Resource Type: Dataset. DOI: https://doi.org/10.22002/spsjf-d1c53

## References

[R1] Clare Elizabeth L., Economou Chloe K., Bennett Frances J., Dyer Caitlin E., Adams Katherine, McRobie Benjamin, Drinkwater Rosie, Littlefair Joanne E. (2022). Measuring biodiversity from DNA in the air. Current Biology.

[R2] Dunham K. 2024. Using airborne eDNA to assess hemlock woolly adelgid ( *Adelges tsugae* ) infestations and their impact on Michigan coastal forests. Masters Thesis. Grand Valley State University.

[R3] Ellison Aaron M., Barker‐Plotkin Audrey A., Foster David R., Orwig David A. (2010). Experimentally testing the role of foundation species in forests: the Harvard Forest Hemlock Removal Experiment. Methods in Ecology and Evolution.

[R4] Evans A. M., Gregoire T. G. (2006). The tree crown distribution of hemlock woolly adelgid,
*Adelges tsugae*
(Hem., Adelgidae) from randomized branch sampling. Journal of Applied Entomology.

[R5] Fidgen Jeffrey G., Whitmore Mark C., Turgeon Jean J. (2015). Ball sampling, a novel method to detect
*Adelges tsugae*
(Hemiptera: Adelgidae) in hemlock (Pinaceae). The Canadian Entomologist.

[R6] Fidgen Jeffrey G., Fournier Ronald E., Whitmore Mark C., MacQuarrie Chris J.K., Turgeon Jean J. (2018). Factors affecting Velcro-covered balls when used as a sampling device for wool of
*Adelges tsugae*
(Hemiptera: Adelgidae). The Canadian Entomologist.

[R7] Fidgen Jeffrey G., Whitmore Mark C., MacQuarrie Chris J.K., Turgeon Jean J. (2021). Detection of
*Adelges tsugae*
(Hemiptera: Adelgidae) wool using Velcro-covered balls. The Canadian Entomologist.

[R8] Havill NP, Montgomery ME, Keena M, Onken B, Reardon R. 2011. Hemlock woolly adelgid and its hemlock hosts: a global perspective. *Implementation and status of biological control of the hemlock woolly adelgid. * US Forest Service, Publication FHTET-2011-04, Morgantown, WV, 3-14.

[R9] Johnson Mark, Barnes Matthew A. (2024). Macrobial airborne environmental DNA analysis: A review of progress, challenges, and recommendations for an emerging application. Molecular Ecology Resources.

[R10] Johnson Mark D., Barnes Matthew A., Garrett Nina R., Clare Elizabeth L. (2023). Answers blowing in the wind: Detection of birds, mammals, and amphibians with airborne environmental DNA in a natural environment over a yearlong survey. Environmental DNA.

[R11] Johnson Mark D., Fokar Mohamed, Cox Robert D., Barnes Matthew A. (2021). Airborne environmental DNA metabarcoding detects more diversity, with less sampling effort, than a traditional plant community survey. BMC Ecology and Evolution.

[R12] Kirtane Anish, Dietschler Nicholas J., Bittner Tonya D., Lefebvre Marshall Bigler, Celis Sabrina, O'Connor Katharine, Havill Nathan, Whitmore Mark C. (2022). Sensitive environmental DNA (eDNA) methods to detect hemlock woolly adelgid and its biological control predators
*Leucotaraxis*
silver flies and a
*Laricobius*
beetle. Environmental DNA.

[R13] Lynggaard Christina, Bertelsen Mads Frost, Jensen Casper V., Johnson Matthew S., Frøslev Tobias Guldberg, Olsen Morten Tange, Bohmann Kristine (2022). Airborne environmental DNA for terrestrial vertebrate community monitoring. Current Biology.

[R14] Lynggaard Christina, Frøslev Tobias Guldberg, Johnson Matthew S., Olsen Morten Tange, Bohmann Kristine (2023). Airborne environmental DNA captures terrestrial vertebrate diversity in nature. Molecular Ecology Resources.

[R15] McClure MS. 1987. *Biology and control of hemlock woolly adelgid* (Vol. 851, pp. 1-9). New Haven, CT: Connecticut Agricultural Experiment Station.

[R16] Orwig David A., Foster David R. (1998). Forest Response to the Introduced Hemlock Woolly Adelgid in Southern New England, USA. Journal of the Torrey Botanical Society.

[R17] (2014). Understanding and Developing Resistance in Hemlocks to the Hemlock Woolly Adelgid. Southeastern Naturalist.

[R18] Roger Fabian, Ghanavi Hamid R., Danielsson Natalie, Wahlberg Niklas, Löndahl Jakob, Pettersson Lars B., Andersson Georg K. S., Boke Olén Niklas, Clough Yann (2022). Airborne environmental DNA metabarcoding for the monitoring of terrestrial insects—A proof of concept from the field. Environmental DNA.

[R19] Sanders M. 2021. Developing novel molecular detection techniques for hemlock woolly adelgid ( *Adelges tsugae* ). Masters Thesis. Grand Valley State University. https://scholarworks.gvsu.edu/theses/1033

[R20] Sanders Meg, Tardani Renee, Locher Alexandra, Geller Kathryn, Partridge Charlyn G (2022). Development of Novel Early Detection Technology for Hemlock Woolly Adelgid,
*Adelges tsugae*
(Hemiptera: Adelgidae). Journal of Economic Entomology.

[R21] Spaulding Heather L., Rieske Lynne K. (2010). The aftermath of an invasion: Structure and composition of Central Appalachian hemlock forests following establishment of the hemlock woolly adelgid, Adelges tsugae. Biological Invasions.

[R22] Stadler Bernhard, Müller Thomas, Orwig David (2006). THE ECOLOGY OF ENERGY AND NUTRIENT FLUXES IN HEMLOCK FORESTS INVADED BY HEMLOCK WOOLLY ADELGID. Ecology.

[R23] Tebbe C C, Vahjen W (1993). Interference of humic acids and DNA extracted directly from soil in detection and transformation of recombinant DNA from bacteria and a yeast. Applied and Environmental Microbiology.

